# Audiomotor Integration in Minimally Conscious State: Proof of Concept!

**DOI:** 10.1155/2015/391349

**Published:** 2015-09-03

**Authors:** Antonino Naro, Antonino Leo, Antonino Cannavò, Antonio Buda, Rocco Bruno, Carlo Salviera, Placido Bramanti, Rocco Salvatore Calabrò

**Affiliations:** ^1^IRCCS Centro Neurolesi “Bonino-Pulejo”, Contrada Casazza, SS113, 98124 Messina, Italy; ^2^Otorhinolaryngoiatry Unit, University of Messina, Messina, Italy

## Abstract

Patients suffering from chronic disorders of consciousness (DOC) are characterized by profound unawareness and an impairment of large-scale cortical and subcortical connectivity. In this study, we applied an electrophysiological approach aimed at identifying the residual audiomotor connectivity patterns that are thought to be linked to awareness. We measured some markers of audiomotor integration (AMI) in 20 patients affected by DOC, before and after the application of a repetitive transcranial magnetic stimulation protocol (rTMS) delivered over the left primary motor area (M1), paired to a transauricular alternating current stimulation. Our protocol induced potentiating of the electrophysiological markers of AMI and M1 excitability, paired to a clinical improvement, in all of the patients with minimally conscious state (MCS) but in none of those suffering from unresponsive wakefulness syndrome (UWS). Our protocol could be a promising approach to potentiate the functional connectivity within large-scale audiomotor networks, thus allowing clinicians to differentiate patients affected by MCS from UWS, besides the clinical assessment.

## 1. Introduction

Patients suffering from chronic disorders of consciousness (DOC) show dissociation between the two main components of consciousness, that is, awareness and wakefulness. Indeed, the unresponsive wakefulness syndrome (UWS) patients do not show signs of awareness (with preservation of wakefulness) whereas the minimally conscious state (MCS) individuals show some purposeful behaviors [1, 2]. DOC differential diagnosis relies on awareness assessment through* ad hoc* behavioral scales, such as the Coma Recovery Scale-Revised (CRS-R) [3]. Behavioral impairment could be related to an extensive connectivity disruption within complex corticothalamocortical networks [4–6]. Nevertheless, some patients could be unable to properly react to stimuli for other reasons, such as poor cooperation or cognitive impairment [7]. Hence, specific paradigms aimed at objectifying a possible correlation between wide brain disconnectivity and motor output failure should be fostered. To this end, there is growing evidence regarding auditory-motor integration processes (AMI) in DOC patients, showing residual preservation of the auditory processing, also involving the associative areas [8–12].

In addition, it has been shown that some noninvasive neurostimulation protocol could unmask residual covert connectivity patterns in some DOC patients, including UWS [13]. Recently, paired associative stimulation (PAS) protocol has been employed in shaping the AMI in healthy individuals [14]. PAS is an electrophysiological technique that pairs conditioning stimuli (e.g., visual, sensory, and auditory stimuli, motor imagery, or movements) with transcranial magnetic stimuli (TMS) over the motor cortex [15–17], thus inducing a long-lasting change in cortical excitability probably by means of Hebbian long-term potentiation or depression-like process (LTP, LTD) [18]. Concerning AMI, conditioning auditory stimuli affect the motor cortex excitability [14], whereas acoustic stimuli paired with TMS over the auditory cortex induce tonotopically specific and tone-unspecific auditory cortex plasticity [19]. In addition, speech perception can modulate the motor cortical excitability within hand, lips, and tongue area representation [20–22].

Hence, aim of the current study was to investigate whether it was possible to induce plasticity within the motor system by applying an audiomotor PAS protocol in DOC patients. To this end, we paired a 5 Hz repetitive TMS (rTMS) over the left M1 with a transauricular repetitive electric stimulation (rES) of the right acoustic nerve in a DOC sample and in healthy individuals (HC). We hypothesized that such paired protocol could induce a M1 excitability increase through the recruitment of residual audiomotor pathways, thus allowing us to differentiate MCS (that should show residual connectivity properties) from UWS individuals (who should lack of such properties), besides the clinical assessment.

## 2. Materials and Methods

### 2.1. Subjects

Of the 47 chronic DOC subjects who attended over two years to the Neurorehabilitation Unit of the IRCCS Centro Neurolesi “Bonino-Pulejo” (Messina, Italy), we enrolled 20 patients who met the criteria for vegetative state and MCS diagnosis [2, 23, 24] and the following inclusion criteria: a DOC condition lasting more than 3 months after the brain injury; no other severe neurological or systemic diseases; no critical conditions (i.e., inability to breathe independently and hemodynamic instability); no cortical excitability-modifying drugs assumption beyond L-DOPA and baclofen; absence of epileptic history, pace-maker, aneurysms clips, neurostimulator, brain/subdural electrodes or other electromechanical devices; absence of electroencephalographic (EEG) burst-suppression pattern; presence of long-latency auditory evoked potentials (LLAEP); no lesion of eardrum or external meatus. In addition, we included 10 HC (6 females and 4 males, mean age: 45.3 ± 6.2 years) as control group in the study.

We resumed the clinical and demographic characteristics in Table 1. DOC etiology consisted of postanoxic or posttraumatic brain damage. The neurological examination mainly showed a pattern of spastic tetraparesis. Two neurologists, skilled in DOC diagnosis, independently evaluated the patients through the JFK CRS-R, which was daily administered for 30 days consecutively, at different times, in order to steadily establish the level of consciousness impairment. EEG examination evidenced continuous slowing in theta and/or delta frequency ranges.

Our Research Institute Ethics Committee approved the present study and either the HC or the legal guardian of each patient gave their written informed consent.

### 2.2. Experimental Design

**Figure 1:**
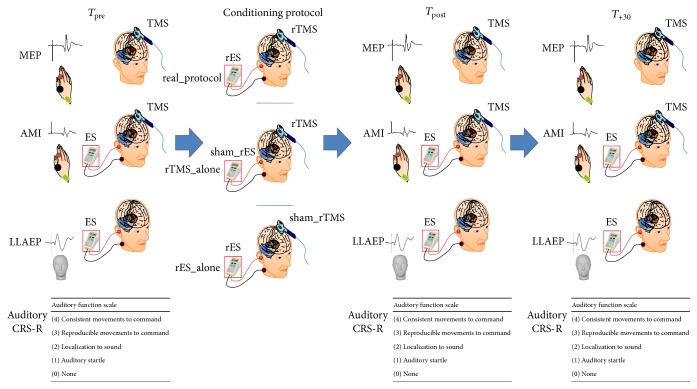
Resuming the experimental design. We measured before (*T*_pre_) and after (*T*_post_ and *T*_+30_) each conditioning protocol (real_protocol, rTMS_alone, and rES_alone) the motor evoked potential (MEP) amplitude, the audiomotor integration (AMI) strength, the long-latency auditory evoked potentials (LLAEP) latency and amplitude, and the CRS-R auditory function.

HC were seated on a comfortable reclining chair, in a mild-lighted room during the entire experimental procedure, whereas the patients were lying in their bed. At baseline (*T*_pre_), we assessed the audiomotor domain score of the CRS-R (in DOC patients), the resting motor threshold (RMT), the motor evoked potential (MEP) peak-to-peak amplitude, the LLAEP latency and amplitude, and the strength of audiomotor interaction (AMI). Then, each participant underwent three different protocols, administered in a random scheme at one-day interval: (i) a real_protocol (rTMS paired to rES); (ii) a rTMS_alone (i.e., rTMS paired to a sham_rES); and (iii) a rES_alone (i.e., rES paired to a sham_rTMS). We repeated the aforementioned baseline measures immediately (*T*_post_) and 30 minutes after (*T*_+30_) the application of each conditioning protocol. The experimental design is summarized in Figure 1. The experimenters who analyzed the data were blinded on the scheme procedure.

### 2.3. Clinical Assessment

The JFK CRS-R is a reliable and standardized scale that integrates neuropsychological and clinical assessment; it includes the current diagnostic criteria for coma, VS, and MCS and allows the clinician to assign a patient to the most appropriate diagnostic category. Hence, the CRS-R represents a good approach for characterizing the level of consciousness and for monitoring the neurobehavioral function recovery [24].

### 2.4. Motor Evoked Potentials

We positioned the coil over the optimum position (hot-spot) to elicit a stable MEP of 0.5 mV peak-to-peak amplitude in the right first dorsal interosseous (FDI) muscle at rest. The hot-spot was identified by moving the coil in 0.5 cm steps around the presumed hot-spot. The coil was held tangentially to the scalp, with the handle pointing backwards and laterally to 45° from the midline (approximately perpendicular to the line of the central sulcus). We thus estimated the RMT, which was defined as the minimum intensity able to evoke a peak-to-peak MEP amplitude of 50 *μ*V in at least five-out-of-ten consecutive trials in the relaxed FDI muscle [25]. Therefore, fifteen MEPs were recorded from the right FDI muscle at rest (using a stimulation intensity of 120% of RMT) at baseline (*T*_pre_), immediately (*T*_post_), and 30 minutes after (*T*_+30_) the application of each conditioning protocol. The peak-to-peak amplitude of each MEP was measured offline, and the mean amplitude was calculated. MEP amplitude changes were calculated as percent of the baseline MEP (*T*_pre_).

We used a high-power Magstim 200 stimulator (Magstim, Whitland, Dyfed, UK) and a standard figure-of-eight coil, with external loop diameters of 9 cm. The magnetic stimuli had monophasic pulse configuration and a rise-time of ~100 *μ*s, decaying back to zero over ~800 *μ*s. The coil current during the rising phase of the magnetic field flowed toward the handle. Thus, the induced current in the cortex flowed in a posterior-to-anterior direction.

### 2.5. Long-Latency Auditory Evoked Potentials

Since a standard AEP assessment in DOC patients is extremely challenging owing to the low and inconsistent cooperation, we chose a rES approach [26] in order to elicit LLAEP. We used a battery-driven stimulator (Brain Stim, E.M.S., Bologna, Italy) with a couple of silver electrodes. The stimulation electrode (a silver ball) was placed in the right external auditory meatus near the eardrum (after having flushed the external auditory meatus with physiologic saline solution) and the reference electrode (a silver disk) on the skin of the patient's neck (near the right mastoid). We delivered two consecutive trains of 200 electric stimuli (500 Hz sine tones at an intensity of 500 *μ*A, at 5 Hz). The intertrain interval was 30 sec. The stimulation procedure induced a hearing sensation of intermediate loudness in the HC. Each participant wore an earplug in the left ear. During the stimulation, we recorded the EEG from electrode Cz referring to the right mastoid using Ag/AgCl electrodes. An electrode at the centre of forehead served as ground. Two additional channels were employed for the electrooculogram (active electrode on the left supraorbital position and the reference electrode on the left infraorbital position). Impedance was ≤10 kΩ. Signals were digitized (A/D = 1000 Hz), amplified (1000 times), and filtered (0.15–100 Hz, 50 Hz-notched) through a 1401 plus AD laboratory interface (Cambridge Electronic Design, Cambridge, UK) and a Digitimer D360 (Digitimer Ltd., Welwyn Garden City, UK) and stored on a personal computer for offline analysis (Signal software, Cambridge Electronic Design, UK). Then, data were processed by artifact rejecting (±100 *μ*V and by subtracting ocular artifacts), epoch from −100 to 500 ms, filtered (1–30 Hz, 12 dB/octave) and averaged. Hence, we registered a cortical triphasic positive-negative-positive potential (P1-N1-P2), starting at around 50 ms in the HC, in analogy to previous LLAEP findings [27, 28]. We measured the component latencies and the baseline-peak amplitude of N1. Latencies were determined by using a modified box-plot method known as the median rule.

### 2.6. Audiomotor Integration

In analogy to a previous work [14], we applied pairs of stimuli consisting of a conditioning stimulus (500 Hz sine tone burst) followed by a magnetic test (90% of AMT), with an interstimulus interval of subject's N1 peak-latency +50 ms [20, 29, 30]. Although it has been reported that speech sounds topographically activate the motor cortex (e.g., [21]), others suggest that the motor cortex might be also nontopographically activated by nonspeech sounds [31]. We registered 15 MEP (test MEP) intermingled with 15 electric-magnetic pairs of stimuli interactions (conditioned MEP) in a single trial, delivered at a frequency of 0.2 Hz at baseline (*T*_pre_) and immediately (*T*_post_) and 30 minutes after (*T*_+30_) the application of each conditioning protocol. We measured the mean amplitude of the conditioned MEP as percentage of the amplitude of the unconditioned MEP (test MEP), which was taken as a measure of the strength of AMI.

### 2.7. rTMS and rES

rTMS was employed in either the real_protocol or the rTMS alone. We delivered 600 stimuli at a frequency of 5 Hz (3 blocks of 200 pulses in 40 seconds, intertrain interval of 10 seconds). The intensity of magnetic stimulation was set at 90% of RMT. For the sham_rTMS, we used the same abovementioned set-up, but with a sham coil. Each rTMS protocol was carried out in accordance with published safety recommendations [32].

Repetitive magnetic stimuli were delivered through a figure-of-eight coil connected to a Magstim Rapid stimulator (Magstim Company, Whitland, Dyfed, UK), with a biphasic waveform of the magnetic stimulus and a pulse width of ~300 *μ*s. The coil was positioned over the hot-spot for the right FDI muscle. During the first phase of the biphasic stimulus, the current flowed in the coil toward the handle and induced a posterior-anterior current within the brain. EMG activity of the right FDI muscle was continuously monitored through loudspeakers throughout the entire rTMS session.

rES was employed in either the real_protocol or the rES alone. It consisted of 600 bursts of 500 Hz sine tone at 5 Hz (3 blocks of 200 pairs in 40 seconds, intertrain interval of 10 seconds) in the right ear, delivered through the aforementioned battery-driven stimulator. With regard to the sham_rES, the electric stimulator was switched off after 30 sec.

### 2.8. Conditioning Protocols

Each participant underwent three different conditioning protocols, administered in a random scheme (i, ii, and iii) and in different sessions, at one-day interval:

- The real_protocol, which consisted of rTMS paired to rES, thus delivering 600 pairs of electric-magnetic stimuli at a frequency of 5 Hz, with an interstimulus interval of subject's N1 latency +50 ms (as in AMI).
- The rTMS_alone (i.e., rTMS paired to a sham_rES), in which the electric stimulator was switched off after 30 sec (thus 600 pairs of sham electric stimuli and real TMS pulses).
- The rES_alone (i.e., rES paired to a sham_rTMS), in which we used a sham_rTMS coil (thus 600 pairs of real electric stimuli and sham TMS pulses).

### 2.9. Statistical Analysis

We compared the baseline clinical and electrophysiological parameters among HC, MCS patients, and UWS patients, through unpaired *t*-tests (calculated on the mean of the three *T*_pre_ values). We thus evaluated the effects of the conditioning protocols on each electrophysiological variable (RMT%, MEP amplitude, AMI strength, and LLAEP latency and amplitude) through separated three-way repeated-measure analyses of variance (rmANOVA), implying* time* (three levels: *T*_pre_, *T*_post_, and *T*_+30_) and* protocol* (three levels: real_protocol, rTMS_alone, and rES_alone), as within-subject factors, and* group* (three levels: MCS patients, UWS patients, and HC) as between-subject factor. The effect of the conditioning protocols on audiomotor CRS-R was measured through a Wilcoxon test. The Greenhouse-Geisser method was used if necessary to correct for nonsphericity. Conditional on a significant *F* value, we performed* post hoc t*-tests (Bonferroni) to explore the strength of main effects and the patterns of interaction between the experimental factors. All statistical tests were applied two-tailed. A significant *p* value was <0.05. All data are given as means or percent changes ±se. We calculated a Spearman correlation test in order to assess an eventual correlation among clinical and electrophysiological parameters.

## 3. Results

We did not observe any side effect in both the patients and HC, either during or after the entire experimental procedure.

### 3.1. DOC/HC Clinical and Electrophysiological Differences at Baseline

We resumed the DOC sample demographic characteristics and the monthly CRS-R scores in Table 1. There were no significant MCS-UWS differences concerning the demographic characteristics, except for slightly longer disease duration in the MCS than the UWS patients. Instead, the monthly and daily CRS-R scores were significantly higher in the MCS than the UWS individuals (≤7). Daily CRS-R scores in each patient showed a relatively low variability during the 30-day observation period. The auditory CRS-R score at each *T*_pre_ was superimposable to the monthly CRS-R score in each patient. Similarly, the baseline electrophysiological parameters were similar and stable during the three days of experimentation. We reported the raw values of the electrophysiological parameters at *T*_pre_ (calculated as mean of the three *T*_pre_ values) for each participant in Table 2. RMT and MEP amplitudes were similar in the three groups. The LLAEP amplitude was slightly reduced only in the UWS individuals, whereas LLAEP latency was significantly increased in the DOC participants (more in the UWS than the MCS patients). The stimulation set-up we used to elicit AMI induced clear inhibitory effects on MEP amplitude in the HC, but such effects were reduced in the MCS patients and nearly absent in the UWS patients.

### 3.2. Conditioning Protocol's Effects on Clinical Assessment

The Wilcoxon test showed a statistically significant increase of the audiomotor CRS-R score only in the MCS patients after the real_protocol at *T*_post_ (*p* = 0.04). Indeed, five MCS patients (numbers 3, 6, 7, 8, and 10) upgraded from a *T*_pre_ “auditory startle” response (1 point at the CRS-R auditory function scale) to a “localization to sound” (2 points) at *T*_post_ (Table 1).

### 3.3. Conditioning Protocol Electrophysiological Effects

**Figure 2:**
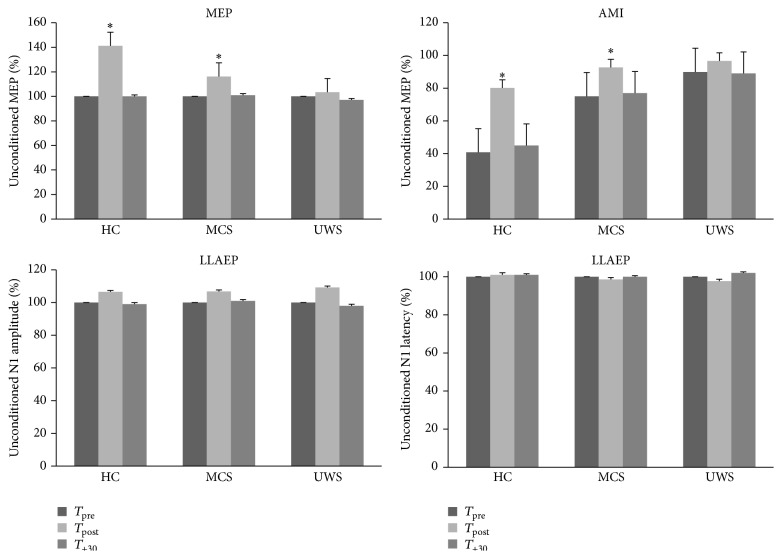
It shows the electrophysiological posteffects induced by the real_protocol. We observed a significant, although short lasting, increase of MEP and AMI amplitude in the HC and MCS patients, whereas the UWS patients did not show any significant effect. The latency and amplitude of LLAEP were affected in none of the groups. The values are reported as mean of the percent change in comparison to the baseline value. The error bars refer to the se. *∗* indicates a significant change (*p* < 0.05).

We resumed in Table 2 and in Figure 2 the time course of electrophysiological parameters following each protocol. We summarized the data statistical analysis in Table 3. The RMT and LLAEP latency and amplitude did not significantly vary after each conditioning protocol. MEP and AMI amplitude significantly increased only in the HC and MCS patients after the real_protocol at *T*_post_. Instead, the *T*_+30_ values were comparable to *T*_pre_ (Figure 2). Notably, none of the UWS patients showed any protocol-induced effect (Figure 2). There were no significant differences concerning the protocol posteffects in relation to the clinical and demographic characteristics. Interestingly, we observed a correlation trend between audiomotor CRS-R amelioration and AMI modulation at *T*_post_ (*r* = 0.576, *p* = 0.07).

## 4. Discussion

For the first time ever, we assessed the presence of residual audiomotor functional plasticity in a DOC sample by means of an audiomotor PAS. Only the real_protocol (rTMS + rES) induced strengthening of the M1 excitability (MEP amplitude increase) and a modification of audiomotor functional connectivity (weakening of inhibitory AMI) in the HC and MCS patients. Such posteffects were paralleled by a transient audiomotor CRS-R score improvement in some MCS patients (i.e., from “auditory startle” to “sound localization”). On the contrary, the UWS patients did not show any clear posteffect.

The clinical and electrophysiological ameliorations in HC and MCS patients mainly depended on the type of the conditioning protocol that was employed, as also previously shown in healthy individuals [14, 19]. In fact, neither the rTMS_alone nor the rES_alone induced any significant posteffect. Indeed, PAS has been suggested to induce associative LTP or LTD-like neuronal synapses via mechanisms of spike-timing dependent synaptic plasticity [18]. Therefore, in our patients, the real-protocol modulated the audiomotor connectivity probably through time-locked neural activity encompassing the primary auditory area and M1. It has been hypothesized that plasticity and connectivity recovery in individuals suffering from DOC might depend on the modulation of postischemic LTP, the production of specific neurotrophins, and the regulation of excitatory/inhibitory dynamics within corticothalamocortical circuits [33–37]. Thereby, it is conceivable that one or more of these mechanisms may have been triggered by the real_protocol and could have favored the recruitment of silent or stunned residual corticothalamocortical projections, thus enhancing the behavioral output in some of our patients. To this end, we could hypothesize the enrolment of a wide audiomotor network including multiple and interconnected cortical areas (encompassing primary auditory cortex, motor areas, and prefrontal cortex) and probably other cortical and subcortical areas (maybe the cerebellum and the basal ganglia) [38–40]. Such network could hierarchically organize different audiomotor processes, thus allowing a repertoire of audiomotor responses ranging from protective reflex motor activations to complex feedback and feedforward processes regarding purposeful motor responses [38, 41–49].

We can therefore argue that the enhancement of the audiomotor clinical responses in the MCS patients could express a functional upgrading, although transient, of the residual brainstem-thalamocortical and corticocortical networks supporting AMI processes, so as to get a higher and more complex motor behavior. On the other hand, our data further confirm the connectivity impairment affecting UWS individuals within audiomotor integration pathways [1, 8]. Nevertheless, the presence of residual functional connectivity in some UWS patients has been evidenced within other sensory-motor modalities (e.g. [13]), thus allowing us to suppose a condition of functional locked-in syndrome [7, 50]. Hence, such issue needs to be further clarified in more detailed audiomotor integration studies.

Notably, we have to highlight other issues concerning the physiological effects of our combined real_protocol:

1. Maladaptive plasticity phenomena could play an important role in limiting the range of our posteffects in all of the UWS and in some MCS patients [51].
2. In the pioneering work of Sowman and coworkers [14], the authors applied speech sound stimuli paired to TMS, being therefore the posteffects potentially dependent on phonological motor resonance and tonotopic-topographic specificity [20, 29], as also suggested by a recent study employing 1–4 kHz tones paired to primary auditory area rTMS [19]. Instead, we triggered brain networks with different tonotopic specificity, whereas the topographic specificity should be more deeply investigated (e.g., by studying the muscle involved in articulation).
3. Since RMT, LLAEP, and MEP amplitude were not substantially different at baseline between HC and DOC and RMT and LLAEP did not vary after the conditioning protocols, we can exclude the possibility that baseline cortical excitability or LLAEP differences could have influenced our posteffects.
4. We may exclude differences in the attentive level in the HC participants in reason of their blinded condition concerning the different experimental sessions [52].
5. The lack of rTMS_alone posteffects on MEP amplitude confirms the findings of a previous high-frequency PAS study in healthy individuals, in which 600 magnetic stimuli failed in producing a significant corticospinal excitability modulation [53]. Therefore, the heterologous sensory stimulation we employed (rTMS + rES) boosted up the cortical effects of rTMS, similarly to previous rapid PAS reports [53, 54].

The relatively small sample size and the consequent mixed etiology represent the main limiting factor in our study. Nonetheless, it is difficult to study a large sample of patients with DOC, since the negative outcome of such patients is still unfortunately high.

## 5. Conclusions

In our opinion, the present study shows a promising approach in an attempt to identify residual patterns of AMI in patients affected by severe DOC. Indeed, our data further support the importance of diagnostic approaches that are independent from patient's cooperation, aimed at assessing the brain connectivity patterns, whose impairment is proportionally related to the awareness impairment. In addition, the possibility to identify such partially preserved corticocortical and corticosubcortical networks in DOC may be useful in the selection of candidate patients for therapeutic and rehabilitative trials by means of noninvasive neurostimulation approaches.

## Figures and Tables

**Table 1:** The clinical and demographic characteristics of the whole sample. We reported the monthly individual and group CRS-R scores ± SD (the CRS-R was daily administered for 30 consecutive days before protocol enrollment), with the unpaired *t*-test values. The MCS patients who ameliorated at the auditory function (*n*. 3, 6, 7, 8, and 10) at *T*_post_ following the real_protocol are marked in bold.

DOC	Gender	Etiology	Age	BI	MRI	CRS-R						
						Total	A	V	M	OM	C	Ar
MCS (*n* = 10)	F	A	72	6	WMH	19 ± 1.5	4 ± .6	4 ± 1.5	5 ± 1.3	2 ± .7	1	3 ± .7
	M	T	51	18	WMH, RBG_h	18 ± .9	3 ± .7	3 ± 1.1	7 ± .9	1 ± 1.4	1	3 ± .7
	**F**	**A**	**66**	**9**	**WMH**	**11 ± .8**	**1 ± 1.1**	**3 ± .8**	**2 ± .9**	**1 ± 1.2**	**1**	**3 ± .8**
	F	T	70	22	^L^Fb_h	15 ± 1	3 ± .6	2 ± .7	5 ± 1.6	2 ± 1.6	1	2 ± .9
	M	T	33	8	multiple_h	14 ± 1	2 ± 1.4	2 ± 1	5 ± 1.6	2 ± .9	1	2 ± .7
	**F**	**A**	**41**	**15**	**WMH**	**11 ± 1**	**1 ± .8**	**1 ± 1.3**	**3 ± 1.5**	**2 ± 1.6**	**1**	**3 ± 1**
	**M**	**T**	**35**	**16**	**WMH, ** ^R^ **B** **G**_**h**	**11 ± .9**	**1 ± 1.2**	**1 ± 1.3**	**3 ± .7**	**2 ± 1.6**	**1**	**3 ± .7**
	**F**	**A**	**29**	**17**	**WMH**	**11 ± .9**	**1 ± 1.2**	**1 ± 1.7**	**3 ± .7**	**2 ± 1.6**	**1**	**3 ± .8**
	M	T	23	18	WMH, LBG_h	12 ± 1	2 ± 1.3	1 ± 1.5	3 ± .9	2 ± 1.1	1	3 ± .9
	**F**	**A**	**47**	**14**	**WMH**	**11.9 ± 1**	**11.4**	**3 ± 1.5**	**2 ± .9**	**2 ± 1.3**	**1**	**3 ± .9**
mean ± SD			*47 ± 18*	*14 ± 5*		*24 ± .8*	*4 ± 1.1*	*4 ± 1.1*	*5 ± 1.6*	*1.8 ± .4*	*1*	*3 ± .4*

UWS (*n* = 10)	M	A	53	8	WMH	5 ± .4	1 ± .4	1 ± 1	1 ± 1.5	1 ± 1.2	0	1 ± 1.1
	F	T	26	3	DAI, SAH	4 ± .5	1 ± .5	1 ± 1.6	1 ± 1.8	0 ± 1	0	1 ± 1.1
	F	T	56	8	^R^FP_h	6 ± .9	0 ± .9	2 ± 1.4	2 ± 1.2	1 ± .7	0	1 ± .8
	F	A	62	11	WMH	6 ± .9	1 ± .9	1 ± 1.5	2 ± 1.5	0 ± 1.7	0	2 ± .8
	M	T	51	9	SAH	4 ± .5	1 ± .9	1 ± 1.6	1 ± 1.4	0 ± 1.2	0	1 ± .9
	M	A	69	11	WMH	7 ± .8	1 ± .5	1 ± 1.9	2 ± 1	1 ± .9	0	2 ± 1.2
	F	T	74	12	DAI, SAH	6 ± .9	1 ± .8	2 ± 1.7	1 ± 1	0 ± 1.9	0	2 ± 1.6
	M	A	69	13	WMH	7 ± 1	1 ± .9	2 ± 1.2	2 ± .9	0 ± 1.3	0	2 ± 1.6
	F	T	44	14	DAI, SAH	7 ± 1	1 ± .9	2 ± 1.7	2 ± 1.3	0 ± 1.8	0	2 ± .9
	F	T	52	15	^R^FT_h	7 ± 1	1 ± .9	2 ± .9	2 ± 1.3	0 ± 1	0	2 ± 1.6
mean ± SD			*56 ± 14*	*10 ± 4*		*5.9 ± 1.1*	*0.9 ± .3*	*1.5 ± .5*	*1.6 ± .5*	*0.3 ± .4*	*0*	*1.6 ± .5*

Unpaired *t*-test	NS	NS	NS	0.03	NS	<0.001	0.02	NS	0.001	<0.001	<0.001	<0.001

Age in years; BI: brain injury onset in months; CRS-R: Coma Recovery Scale-Revised including auditory (A), visual (V), motor (M), oromotor (OM), communication domain (C), and arousal induction (Ar); etiology: A, postanoxic; T, posttraumatic brain injury; MRI: structural patterns including WMH (white matter hyperintensity), _h (hemorrhagic lesion), ^R^FP (right frontopolar), ^R^BG (basal ganglia), ^L^Fb (left frontobasal), SAH (subarachnoid hemorrhage), and DAI (diffuse axonal injury); SD: standard deviation.

**(a):** 

Parameter	Protocol	HC			MCS patients			UWS patients		
		*T*_pre_	*T*_post_	*T*_+30_	*T*_pre_	*T*_post_	*T*_+30_	*T*_pre_	*T*_post_	*T*_+30_
RMT (%)	rES_alone	58	52	52	58	56	58	58	62	67
		52	57	58	60	57	55	68	59	57
		53	57	57	59	54	58	57	60	53
		55	55	54	56	60	55	60	62	57
		58	58	54	59	55	56	66	59	56
		52	56	55	56	58	59	55	66	67
		53	55	53	58	60	57	57	55	65
		56	52	53	57	58	59	66	62	60
		56	55	52	56	60	56	53	60	61
		57	55	53	57	59	58	64	63	54
		*55 ± .7*	*55 ± .6*	*54 ± .6*	*58 ± .5*	*58 ± .7*	*57 ± .5*	*60 ± 1.7*	*61 ± .9*	*60 ± 1.6*
	rTMS_alone	54	54	56	57	54	60	68	53	65
		53	56	53	57	55	58	63	63	65
		58	52	57	58	59	57	67	55	53
		55	58	53	59	59	55	66	67	60
		53	55	58	54	55	57	64	61	58
		52	53	53	55	55	59	60	65	56
		55	57	55	56	56	57	62	55	56
		56	53	55	54	58	60	63	61	59
		53	56	58	58	54	57	56	66	58
		56	54	52	59	56	57	53	54	59
		*55 ± .6*	*55 ± .6*	*55 ± .7*	*57 ± .6*	*56 ± .6*	*58 ± .5*	*62 ± 1.5*	*60 ± 1.7*	*59 ± 1.2*
	real_protocol	53	52	52	60	56	57	60	58	62
		58	56	58	54	56	56	57	53	67
		58	57	56	**59**	**54**	**59**	61	58	56
		58	57	55	58	54	59	54	64	55
		53	54	53	60	55	60	61	60	69
		56	52	52	**56**	**60**	**57**	56	53	61
		53	58	57	**57**	**56**	**60**	66	60	64
		52	57	58	**58**	**59**	**57**	58	60	57
		56	56	55	59	54	56	65	67	67
		56	53	52	**58**	**58**	**59**	57	61	57
		*55 ± .8*	*55 ± .7*	*54 ± .8*	*57 ± .6*	*56 ± .7*	*58 ± .5*	*59 ± 1.2*	*59 ± 1.4*	*61 ± 1.6*

MEP (mV6)	rES_alone	0.3	0.3	0.3	0.5	0.5	0.7	0.6	0.6	0.5
		0.6	0.6	0.6	0.6	0.6	0.7	0.5	0.5	0.6
		0.9	0.9	0.9	0.6	0.6	0.6	0.4	0.5	0.5
		0.8	0.8	0.8	0.6	0.6	0.7	0.5	0.5	0.5
		0.9	0.9	0.9	0.6	0.7	0.5	0.6	0.4	0.5
		0.9	0.9	0.9	0.7	0.6	0.6	0.6	0.5	0.5
		0.8	0.8	0.8	0.6	0.5	0.7	0.6	0.5	0.6
		0.8	0.8	0.8	0.7	0.5	0.7	0.5	0.5	0.5
		0.8	0.8	0.8	0.6	0.5	0.5	0.5	0.6	0.5
		0.8	0.8	0.8	0.7	0.5	0.6	0.6	0.5	0.5
		*0.7 ± .1*	*0.7 ± .1*	*0.7 ± .1*	*0.6 ± 0.02*	*0.6 ± 0.02*	*0.6 ± 0.02*	*0.5 ± 0.02*	*0.5 ± 0.02*	*0.5 ± 0.02*
	rTMS_alone	0.3	0.3	0.3	0.7	0.5	0.7	0.5	0.5	0.6
		0.6	0.6	0.6	0.6	0.6	0.5	0.4	0.5	0.5
		0.9	0.9	0.9	0.7	0.5	0.7	0.6	0.5	0.5
		0.8	0.8	0.8	0.7	0.6	0.6	0.6	0.5	0.5
		0.9	0.9	0.9	0.6	0.6	0.6	0.6	0.6	0.6
		0.9	0.9	0.9	0.5	0.6	0.6	0.6	0.6	0.4
		0.8	0.8	0.8	0.7	0.6	0.5	0.4	0.6	0.4
		0.8	0.8	0.8	0.6	0.5	0.7	0.5	0.4	0.4
		0.8	0.8	0.8	0.6	0.6	0.5	0.5	0.6	0.4
		0.8	0.8	0.8	0.7	0.6	0.6	0.5	0.5	0.5
		*0.7 ± .1*	*0.7 ± .1*	*0.7 ± .1*	0.6* ± .1*	0.6* ± .1*	*0.6 ± .1*	*0.5 ± 0.02*	*0.5 ± 0.02*	*0.5 ± 0.02*
	real_protocol	0.3	0.7	0.3	0.8	0.8	0.8	0.5	0.6	0.5
		0.6	0.8	0.6	0.9	0.9	0.9	0.5	0.5	0.5
		0.9	1.2	0.9	**0.6**	**0.8**	**0.6**	0.4	0.6	0.4
		0.8	1.0	0.8	0.8	0.9	0.8	0.6	0.4	0.4
		0.9	1.2	0.9	0.8	0.8	0.8	0.4	0.5	0.5
		0.9	1.2	0.9	**0.9**	**1**	**0.9**	0.4	0.5	0.5
		0.8	1.1	0.8	**0.8**	**1**	**0.8**	0.5	0.5	0.5
		0.8	1.1	0.8	**0.3**	**0.9**	**0.3**	0.6	0.5	0.5
		0.8	1.2	0.8	0.8	0.8	0.8	0.5	0.5	0.4
		0.8	1.1	0.8	**0.9**	**1**	**0.9**	0.5	0.5	0.6
		*0.7 ± .1*	*1.1 ± .1*	*0.7 ± .1*	*0.7 ± .1*	*0.9 ± .05*	*0.7 ± .1*	*0.5 ± 0.02*	*0.5 ± 0.02*	*0.5 ± 0.02*

AMI (mV)	rES_alone	0.5	0.4	0.3	0.6	0.5	0.5	0.6	0.5	0.5
		0.3	0.5	0.4	0.4	0.5	0.5	0.4	0.5	0.5
		0.4	0.5	0.3	0.4	0.5	0.5	0.4	0.5	0.5
		0.5	0.3	0.4	0.5	0.5	0.5	0.5	0.5	0.5
		0.5	0.5	0.3	0.4	0.6	0.5	0.4	0.6	0.5
		0.5	0.3	0.4	0.5	0.4	0.5	0.5	0.4	0.5
		0.3	0.3	0.4	0.4	0.5	0.6	0.4	0.5	0.6
		0.5	0.4	0.3	0.5	0.5	0.4	0.5	0.5	0.4
		0.3	0.4	0.3	0.5	0.6	0.4	0.5	0.6	0.4
		0.5	0.4	0.3	0.5	0.4	0.4	0.5	0.4	0.4
		*0.4 ± 0.02*	*0.4 ± 0.02*	*0.4 ± 0.02*	*0.5 ± 0.02*	*0.5 ± 0.02*	*0.5 ± 0.02*	*0.5 ± 0.02*	*0.5 ± 0.02*	*0.5 ± 0.02*
	rTMS_alone	0.5	0.3	0.4	0.5	0.4	0.5	0.5	0.4	0.5
		0.5	0.4	0.4	0.5	0.5	0.6	0.5	0.5	0.6
		0.4	0.3	0.3	0.5	0.6	0.5	0.5	0.6	0.5
		0.3	0.3	0.4	0.5	0.4	0.5	0.5	0.4	0.5
		0.3	0.5	0.4	0.6	0.4	0.6	0.6	0.4	0.6
		0.3	0.3	0.5	0.5	0.5	0.5	0.5	0.5	0.5
		0.3	0.3	0.4	0.5	0.5	0.4	0.5	0.5	0.4
		0.4	0.3	0.3	0.4	0.5	0.5	0.4	0.5	0.5
		0.4	0.3	0.5	0.6	0.5	0.5	0.6	0.5	0.5
		0.4	0.4	0.5	0.6	0.5	0.5	0.6	0.5	0.5
		*0.4 ± 0.02*	*0.4 ± 0.01*	*0.4 ± 0.02*	*0.5 ± 0.02*	*0.5 ± 0.02*	*0.5 ± 0.02*	*0.5 ± 0.02*	*0.5 ± 0.02*	*0.5 ± 0.02*
	real_protocol	0.5	0.9	0.4	0.6	0.3	0.5	0.6	0.4	0.6
		0.4	1	0.3	0.4	0.6	0.4	0.5	0.4	0.5
		0.3	1	0.4	**0.6**	**0.9**	**0.5**	0.5	0.4	0.4
		0.3	0.8	0.5	0.5	0.8	0.5	0.6	0.6	0.6
		0.4	0.9	0.5	0.4	0.9	0.4	0.5	0.6	0.5
		0.5	0.8	0.3	**0.5**	**0.9**	**0.6**	0.6	0.5	0.4
		0.4	0.8	0.4	**0.4**	**0.8**	**0.5**	0.4	0.5	0.5
		0.4	0.9	0.4	**0.5**	**0.8**	**0.6**	0.5	0.5	0.5
		0.5	1	0.4	0.4	0.8	0.6	0.5	0.5	0.5
		0.4	0.8	0.5	**0.4**	**0.8**	**0.5**	0.5	0.6	0.6
		*0.4 ± .03*	*0.9 ± .05*	*0.4 ± .02*	*0.5 ± 0.5*	*0.7 ± .1*	*0.5 ± 0.02*	*0.5 ± 0.02*	*0.5 ± 0.02*	*0.5 ± 0.02*

LLAEP amplitude (*μ*V)	rES_alone	16	3	7	7	11	11	5	0	2
		6	4	2	11	11	7	4	2	5
		3	6	7	10	10	13	0	7	1
		14	1	8	11	9	6	7	6	2
		2	3	6	6	12	5	2	0	0
		9	10	15	5	11	8	6	7	2
		16	8	15	12	7	12	6	7	6
		10	12	11	12	5	5	5	1	4
		1	5	16	5	13	9	6	6	2
		9	3	16	8	6	10	5	2	6
		*9 ± 1*	*6 ± 2*	*10 ± 2*	*9 ± 1*	*10 ± 1*	*9 ± 1*	*5 ± 1*	*4 ± 0.4*	*3 ± 1*
	rTMS_alone	16	0	2	12	8	7	2	1	7
		7	10	7	13	13	6	3	1	4
		7	6	8	12	11	8	7	7	6
		16	15	10	5	11	5	2	7	4
		14	1	0	8	9	11	4	7	6
		16	8	4	12	8	7	4	6	2
		5	10	11	6	11	13	5	7	4
		5	16	13	5	13	10	6	1	5
		15	9	5	6	9	10	3	4	0
		1	2	2	7	13	13	2	1	4
		*10 ± 2*	*8 ± 2*	*6 ± .4*	*9 ± 1*	*11 ± 1*	*9 ± 1*	*4 ± 1*	*4 ± 1*	*4 ± 1*
	real_protocol	12	3	13	9	9	11	0	0	2
		16	5	6	9	9	7	0	1	4
		6	6	6	**12**	**12**	**10**	7	4	5
		3	16	2	13	11	8	6	2	2
		12	10	2	7	9	12	7	3	6
		4	7	16	**11**	**6**	**5**	2	4	3
		6	11	10	**10**	**5**	**9**	4	5	6
		14	15	8	**13**	**11**	**13**	3	2	2
		3	3	14	10	11	6	3	5	6
		4	14	6	**9**	**5**	**7**	5	6	4
		*8 ± 2*	*9 ± 1*	*8 ± 1*	*10 ± .4*	*9 ± 1*	*9 ± 1*	*4 ± .4*	*3 ± 1*	*4 ± 1*

LLAEP latency (ms)	rES_alone	89	124	100	111	118	114	131	124	123
		81	95	90	98	117	126	162	135	125
		95	127	133	95	115	131	117	118	106
		84	97	110	110	147	135	162	163	121
		89	96	127	111	145	114	161	161	119
		95	105	126	110	130	151	164	126	122
		82	110	88	109	140	140	139	138	110
		79	84	103	102	107	115	130	140	107
		91	109	105	108	132	143	138	133	106
		86	94	103	101	136	128	116	148	108
		*87 ± 2*	*104 ± 4*	*109 ± 5*	*106 ± 2*	*129 ± 4*	*130 ± 4*	*142 ± 3*	*138 ± 5*	*115 ± 6*
	rTMS_alone	89	109	97	113	111	133	126	123	124
		81	86	82	103	98	111	171	125	157
		95	130	106	103	95	105	135	106	146
		84	107	89	148	110	126	132	121	153
		89	95	117	122	111	111	166	119	129
		95	119	104	110	110	115	142	122	147
		82	95	107	132	109	117	146	110	134
		79	104	84	131	102	114	131	107	127
		91	113	105	138	108	128	145	106	126
		86	109	100	135	101	108	113	108	110
		*87 ± 2*	*107 ± 4*	*99 ± 4*	*123 ± 2*	*106 ± 5*	*117 ± 3*	*141 ± 3*	*115 ± 6*	*135 ± 5*
	real_protocol	89	116	117	155	119	111	128	137	159
		81	97	90	124	122	98	106	146	139
		95	96	114	**102**	**110**	**95**	133	110	121
		84	86	96	135	107	110	147	126	128
		89	120	111	139	106	111	142	140	119
		95	121	124	**135**	**122**	**110**	132	146	168
		82	108	106	**111**	**110**	**109**	132	153	125
		79	86	94	**103**	**107**	**102**	103	119	109
		91	97	116	116	106	108	150	123	110
		86	105	97	**117**	**108**	**101**	126	115	132
		*87 ± 2*	*103 ± 4*	*106 ± 4*	*124 ± 2*	*112 ± 5*	*106 ± 5*	*130 ± 2*	*132 ± 5*	*131 ± 6*

**(b):** 

Parameter	*p* HC/DOC	*p* MCS/UWS
RMT (%)	NS	NS
MEP (mV)	NS	NS
AMI (%)	0.002	0.002

N1 latency (ms)	<0.001	0.005
N1 amplitude (*μ*V)	NS	0.05

AMI: audiomotor integration; LLAEP: long-latency auditory potential; MEP: motor evoked potential; NS: nonsignificant; rES: repetitive electric stimulation; RMT: resting motor threshold; rTMS: repetitive transcranial magnetic stimulation.

**Table 3:** We observed significant real_protocol posteffects in the HC and MCS patients concerning MEP amplitude and AMI strength at *T*_post_, whereas the UWS patients did not show any significant posteffect. The rTMS_alone and the rES_alone did not induce any significant posteffect. The nonsignificant interactions, factors, and time intervals are not shown.

	Time × group × protocol interaction *F*_(8,216)_, *p*	Time × protocol interaction *F*_(4,36)_, *p*		Time effect			
				*F*_(2,18)_, *p*		*t*_(1,9)_, *p*	
MEP amplitude	22, <0.001	HC	90, <0.001		90, <0.001		3.5, 0.001
		MCS	53, <0.001	real_protocol	12, <0.001	*T*_0_	3.6, 0.001
AMI %	13, <0.001	HC	78, <0.001		78, <0.001		3.5, 0.002
		MCS	6.3, 0.006		9.4, 0.001		2.4, 0.02
